# 1-MNA Ameliorates High Fat Diet-Induced Heart Injury by Upregulating Nrf2 Expression and Inhibiting NF-κB *in vivo* and *in vitro*

**DOI:** 10.3389/fcvm.2021.721814

**Published:** 2021-10-12

**Authors:** Ziguang Song, Xiao Zhong, Mingyang Li, Pingping Gao, Zhongping Ning, Zhiqi Sun, Xiang Song

**Affiliations:** ^1^Cardiovascular Center, The Fourth Affiliated Hospital, Harbin Medical University, Harbin, China; ^2^Department of Cardiovascular Medicine, Shanghai University of Medicine & Health Sciences Affiliated Zhoupu Hospital, Shanghai, China; ^3^Department of Cardiovascular Medicine, DaQing Oilfield General Hospital, Daqing, China

**Keywords:** 1-methylnicotinamide, free fatty acids, inflammation, apoptosis, fibrosis, Nrf2, NFkB

## Abstract

High levels of free fatty acids (FFA) are closely associated with obesity and the development of cardiovascular diseases. Recently, nicotinamide adenine dinucleotide (NAD) metabolism has emerged as a potential target for several modern diseases including diabetes. Herein, we explored the underlying mechanisms of NAD metabolism associated with the risk of cardiovascular disease. Our study found that nicotinamide N-methyltransferase (NNMT) mRNA levels were significantly increased in the hearts of FFA-bound-albumin-overloaded mice and in H9C2 cells treated with palmitic acid (PA). We studied the mechanisms underlining the anti-inflammatory and anti-oxidant activities of 1-methylnicotinamide (1-MNA), a metabolite of NNMT. We found a significantly higher level of reactive oxygen species, inflammation, apoptosis, and cell hypertrophy in PA-treated H9C2 cells and this effect was inhibited by 1-MNA treatment. *in vivo*, 1-MNA improved inflammation, apoptosis, and fibrosis damage in mice and this inhibition was associated with inhibited NF-κB activity. In conclusion, our study revealed that 1-MNA may prevent high fatty diet and PA-induced heart injury by regulating Nrf2 and NF-κB pathways.

## Introduction

Elevated plasma free fatty acid (FFA) levels have emerged as a major link between obesity, metabolic syndrome and cardiovascular diseases. Circulating free fatty acids (FFA), mainly originating from lipolysis in the adipose tissue, has been recognized as one of the most important factors causing systemic organ damage to the heart, liver, and skeletal muscle. These adverse effects are defined lipotoxicity (1). Elevated plasma FFA levels induce chronic inflammation, cardiovascular disease, and insulin resistance (2, 3).

Increased levels of FFA promote the expression of pro-inflammatory mediators, such as TNF-α, IL-1, and IL-6. These pro-inflammatory factors further induce oxidative stress. FFA activates the NF-κB pathway (4) and may also induce cell apoptosis and damage (5). It has been reported that cellular redox is closely related to high levels of palmitic acid (PA), which generates excessive lipid-derived free radicals. Studies have shown that a high-fat diet (HFD) results in elevated blood FFA levels and induces inflammation and oxidative stress in various organs including the heart, which subsequently leads to fibrosis, cell apoptosis, and heart injury (6, 7). Anti-inflammatory and antioxidant therapies, therefore, may have important protective and antagonistic effects on injury induced by FFA.

Nicotinamide adenine dinucleotide (NAD) is a coenzyme for redox reactions in eukaryotes and plays an important role in the occurrence and development of cell apoptosis and redox reactions (8). In mammals, key molecules of NAD metabolism regulate various physiological processes (9). For instance, 1-methylnicotinamide (1-MNA) is an effective treatment for refractory hyperproteinuria as it reduces lipid-mediated oxidative stress and cell damage (10). Under Regulation 2015/2283 of the European Parliament and Council, and the European Commission issued Regulation 2018/1123, 1-MNA chloride has been authorized as a dietary supplement.

Experimental treatment of 1-MNA has shown promise in some diseases (11). However, the protective effect of 1-MNA on FFA and HFD-induced heart injury is still unclear. In this study, we explored the anti-inflammatory and anti-oxidant activity of 1-MNA *in vitro* using the cardiomyocyte H9C2 cell line and *in vivo* using a mouse model of hyperlipidemia. Our results showed that 1-MNA mitigated PA and HFD-induced heart cardiac hypertrophy, apoptosis, and myocardial fibrosis. This effect occurred through the activation of Nrf2 and inhibition of the NF-κB pathway.

## Materials and Methods

### Materials

PA and 1-MNA were purchased from Sigma-Aldrich (St. Louis, MO, USA). Stock solutions of 5 mM PA/10% BSA were prepared as follows and the stored at −20°C. Stock solutions were heated at 37°C for 20 min and 1-MNA was dissolved in DMEM and isotonic saline for *in vivo* experiments. Enhanced chemiluminescent reagent kits, Annexin V-FITC apoptosis detection kits, and TUNEL apoptosis detection kits were purchased from Beyotime (Beyotime Biotechnology, Beijing, China). Anti-fluorescence Quenching Mounting Tablets were purchased from SouthernBiotech (0100-01, Birmingham, USA).

### Cell Culture and Measurement of Oxidative Stress (ROS)

The H9C2 embryonic mouse heart cell line was obtained from the Shanghai Cell Bank, Chinese Academy of Sciences and was cultured in DMEM/F12 medium (Gibco, Eggenstein, Germany) with 10% FBS, 2.25 g/L glucose, 100 U/mL penicillin, and 100 mg/mL streptomycin. The H9C2 cell was cultured at an ambient temperature of 37°C and 5% CO2 humid environment. The intracellular ROS level was measured using 2,7-dichloro-dihydro fluorescein diacetate (DCFH-DA). Cells were pretreated with 1-MNA (10 mM) for 6 h, incubated with PA (500 μM) for 12 h. Then, 2 μmol/L (DCFH-DA) was added to cells at 37°C for 30 min. The fluorescence intensity was measured using a fluorescence microscope, at an excitation wavelength of 488 nm. Cells were collected and sorted by flow cytometry (Beckman Coulter) and Cell Quest software for flow cytometry.

### Immunofluorescence Staining

Immunofluorescence was used to measure the cell surface area and apoptosis. To determine apoptosis, the cells were harvested cells and stained with Annexin V and propidium iodide after treatment, and then analyzed by flow cytometry using the sorting flow cytometer and Cell Quest software. For morphology examination and cell surface measurement, the cells were fixed with 4% paraformaldehyde for 20 min in a completely dark environment, washed 3 times with PBS at room temperature. Cells were permeabilized in 0.1% Triton X-100 for 10 min, washed 3 times with PBS at room temperature, and then stained with rhodamine-phalloidin at a concentration of 50 μg/mL for 30 min at room temperature. Specimens were covered with a cover slip and nail polish. The slides were washed 3 times with PBS and subjected to fluorescence microscopy.

In the immunofluorescence study to detect NF-κB, the cells were fixed and permeabilized as indicated above, and the cells were incubated with p65 antibody (1:200) at 4°C overnight, and then incubated with FITC secondary antibody (1:200) at room temperature for 2 h. After each incubation of the antibody, slides were washed 3 times with TBST, 5 min each time. The stained sections were observed under a Nikon fluorescence microscope (Nikon, Japan).

### Animal Model

Eight week-old 18–22 g male C57BL/6 mice were purchased from the Animal Center of the Second Affiliated Hospital of Harbin Medical University. Thirty mice (*n* = 30) were housed in cages in a controlled environment of 22 ± 2.0°C and 50 ± 5% humidity and were maintained on 12-h light/12-h dark cycles with free access to water and food. The mice were divided into two groups. Group I mice (*n* = 10) were fed on normal diet as a control group. Group II mice (*n* = 10) were given a HFD (Hyperlipidemia model feed from Beijing Keao, China). Group III mice were given HFD and a daily gavage of 100 mg/kg/day 1-MNA solution. After eitghteen weeks, all animals were euthanized using CO_2_ and the heart weights were recorded. Blood samples were collected by cardiac puncture as follows. Animals were fixed on their back and with the index finger of the operator's left hand touching the strongest apex of the heart in the third to fourth intercostal space on the left, the right hand used a syringe with a needle to puncture and collect the sample. Blood was mixed with Hank's solution and centrifuge for 15 min at 4°C to collect serum. Mouse hearts were collected under aseptic conditions and heart weights were measured. The tissues used for RNA analysis were snap-frozen and stored at −80°C. The tissues used for histology study were fixed in 10% formalin.

### Western Blotting Analysis

Cultured H9C2 cells or mouse heart tissues were homogenized in RIPA lysis buffer (Beyotime Biotechnology, Beijing, China). Total protein concentrations were determined using a BCA protein concentration kit (Beyotime Biotechnology, Beijing, China). Equal amounts of total protein were analyzed by western blotting. The samples were electrophoresed and electrotransferred to polyvinylidene difluoride membranes (Immobilon; Millipore, Bedford, MA, USA). The membranes were incubated with the indicated primary antibodies and horseradish peroxidase-conjugated anti-rabbit or anti-mouse secondary antibodies (Abcam, Cambridge, UK). The blots were visualized using an enhanced chemiluminescence detection system (Beyotime, Beijing, China). The density of the protein bands was quantified using a gel imaging system (BioRad, Hercules, CA). The following antibodies were used in this study: NRF2 (12721T, CST, Danvers, MA), NQO-1 (sc-376023, Santa Cruz, California, CA),HO-1 (82206S, CST, Danvers, MA), GCLC (ab53179, Abcam, Cambridge, MA), β-actin (3700s, CST, Danvers, MA). BAX (sc-7480, Santa Cruz, California, CA), BCL2 (sc-7382, Santa Cruz, California, CA), Caspase 3 (9662, CST, Danvers, MA), Cleaved Caspase 3 (9664, CST, Danvers, MA), Tgf-β (3711s, CST, Danvers, MA), SMAD3 (9523, CST, Danvers, MA), p-SMAD3 (9520s, CST, Danvers, MA), COL-1 (91144s, CST, Danvers, MA), I kappa B-alpha (sc-1643, Santa Cruz, California, CA), TNF-α (ab183218, Abcam, Cambridge, MA), MMP9 (ab283575, Abcam, Cambridge, MA), and SIRT1 (8469s, CST, Danvers, MA).

### RNA Extraction and Quantitative Real-Time PCR (qRT-PCR)

Total RNA was extracted from tissues and cells using the Trizol method. The cDNA was synthesized using a high-capacity cDNA reverse transcription kit. qRT-PCR was performed on the ABI PRISM 7,500 Sequence Detection System. Primers for NNMT, TNF-α, IL-6, IL-1, TGF-β, Nrf2, heme oxygenase-1 (HO-1), glutamate-cysteine ligase (GCLC), and NADPH quinineoxidoreductase-1 (NQO-1), type 1 collagen, connective tissue growth factor (CTGF), matrix metallopeptidase 9 (MMP-9), α-myosin heavy chain (α-MyHC), brain natriuretic peptide (BNP), and β-actin were purchased from Life Technologies. The mouse housekeeping gene β-actin was used as an internal control. Relative mRNA expression levels were normalized to β-actin. The primer sequences are provided in Supplementary Table 1.

### Histological Analysis

The heart tissues fixed in 10% formalin were dehydrated using gradient alcohol, cleared by xylene, and embedded in paraffin. The sections were cut into slices at a thickness of 7 μm using a Leica RM 2145 microtome (Leica Microsystems, Nussloch, Germany). The paraffin sections were baked in a 60°C oven for 3 h and then de-waxed with xylene 3 times for 20 min in each solution. The tissues were then dehydrated sequentially in absolute ethanol I, absolute ethanol II, 95% ethanol, 90, 80, and 70% alcohol, and finally washed in distilled water for 10 min. Tissues were treated with antigen repair buffer by microwave and washed in PBS (PH 7.4). The first and second antibodies were added successively and incubated at 4°C and 37°C for color development. The nuclear stain DAPI was applied as a counterstain. The slides were air dried and mounted with anti-fluorescence quenching mounting medium, then observed at 400 magnification under fluorescence microscope (Nikon Inc., Japan).

### Measurement of Blood Lipid Levels

Blood lipid levels were determined by using total cholesterol assay kit, triglyceride assay kit and low-density lipoprotein cholesterol assay kit (Nanjing Jiancheng Institute of Bioengineering, China). Briefly, samples, as well as blank distill water and calibrator were mixed with working solution in proportion, then incubated at 37°C for 10 min. Samples were analyzed at wavelength 510 nm, light path 0.5 cm using automatic biochemical analyzer (Chemray240, Rayto, China) to measure the concentration of serum triglyceride (TG), total cholesterol (TC), and low-density lipoprotein (LDL).

### Masson's Trichrome Stain

Paraffin sections were deparaffinized and hydrated in distilled water. The sections were rinsed gently with deionized water blotting the excess liquid on the slides. Weigert's iron hematoxylin staining was applied for 10 min, followed by 1% hydrochloric acid alcohol differentiation. After cleaning, Ponceau acid fuchsin solution staining was applied for 10 min, phosphomolybdic acid aqueous solution treatment for about 5 min, and aniline blue liquid counterstaining was applied for 5 min. Slides were dehydrated with anhydrous ethanol, mounted with anti-quenching mounting plate solution, and then observed under a microscope.

### TUNEL Staining

The cell apoptosis were analyzed uing TUNEL apoptosis detection kits. Paraffin sections are deparaffinized and hydrated in distilled water 100 μl of 20 μg/ml Proteinase K solution were added and incubated at room temperature for 20 min. After washing, 100 μl 1 × Equilibration Buffer were added and incubate at room temperature for 15 min. The tissue sections were incubated with Alexa Fluor 647-12-dUTP Labeling Mix in a wet box at 37°C for 60 min. After washing in PBS, the slides were nuclear counterstained with DAPI for 5 min and mounted with anti-fluorescence mounting solution. The slides were examined under a fluorescence microscope.

### Statistical Analysis

Data are presented as the mean ± SEM. Comparison of multiple groups was performed using analysis of variance (ANOVA) with *post-test* Bonferroni-corrected *t*-test as *post hoc* test. An unpaired Student's *t*-test was used to compare two unmatched groups. Pearson's correlation coefficients were calculated to investigate the association between indicated parameters. *P*-values < 0.05 were considered statistically significant. For graphing and statistical analysis, we used GraphPad Prism (version 8, GraphPad Software Inc., San Diego, CA).

## Results

### Elevated NNMT mRNA Levels in PA-Treated H9C2 Cells

**Figure 1:**
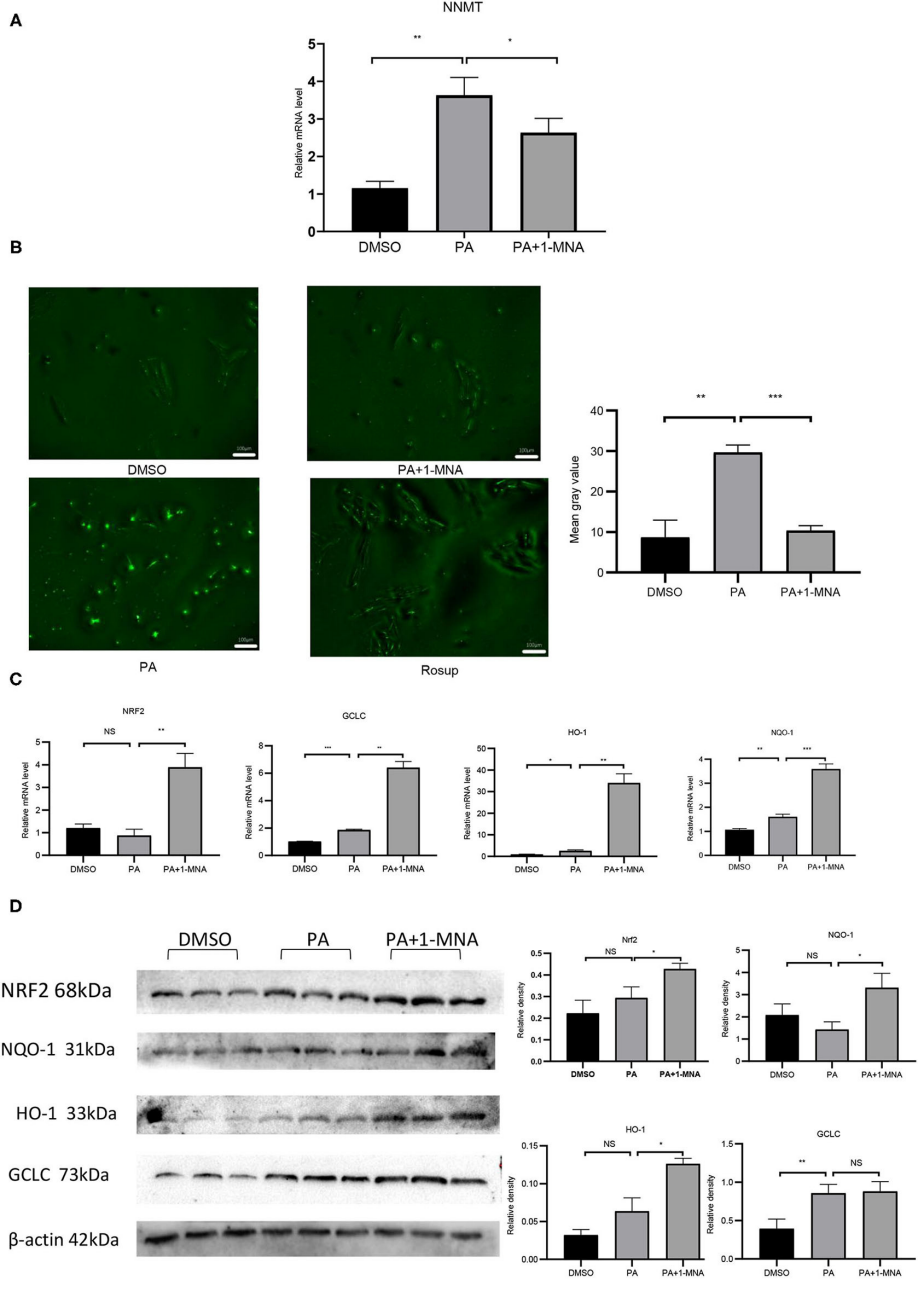
Effects of 1-MNA on PA-induced Oxidative stress in H9C2 cells. **(A)** Real-type quantitative polymerase chain reaction analysis of mRNA expression of NNMT in the H9C2 cells.β-actin was taken as an internal control gene. Expression values are expressed as the mean ± SEM. **(B)** 1-MNA inhibits PA-induced ROS production. Immunofluorescence images of the PA group. The histogram shows the normalized intensity from three independent experiments. **(C)** qRT-PCR analysis of Nrf2, HO-1, GCLC, and NQO-1 expression. **(D)** Western blotting analysis of Nrf2, HO-1, GCLC, and NQO-1. β-actin was taken as an internal control gene. Expression is expressed as the mean ± SEM. Differences in expression were analyzed by the unpaired Student′s *t*-test and one-way ANOVA (*n* = 3, NS = no significance, ^*^p < 0.05, ^**^p < 0.01, ^***^p < 0.001).

NNMT enzyme transfers the methyl group from S-adenosylmethionine (SAM) to nicotinamide resulting in the formation of 1-MNA. To examine the possibility that NNMT is associated with PA-induced heart damage, we first investigated the mRNA expression level of NNMT in cardiomyocyte H9C2 cells. As shown in Figure 1A, the mRNA expression of endogenous NNMT increased 2.4-fold (*p* < 0.01) following PA treatment. 1-MNA prevented PA-induced NNMT expression by 27.4% (*p* < 0.05). This result indicated that NNMT was functionally overexpressed in H9C2 cells and in the PA overload model.

### 1-MNA Prevented ROS Formation and Oxidative Stress in PA-Induced H9C2 Cells

Elevated circulating FFA levels result in cardiovascular complications and oxidative stress. We measured the intracellular ROS level in H9C2 cells treated with PA or PA plus 1-MNA using 2,7-dichloro-dihydro fluorescein diacetate (DCF). As shown in Figure 1B, ROS production and ROS-positive DCFH-DA intensity was 241.4% increased in cells with 500 μM PA treatment than in the control group (*p* < 0.01). This increase was markedly reduced by 1-MNA treatment by 65.1% (*p* < 0.001). Next, we used qRT-PCR and western blotting to study the expression of Nrf2 after the removal of ROS by 1-MNA (Figures 1C,D). In cells treated with 10 mM 1-MNA, mRNA and protein levels of Nrf2 were increased 347.1% (*p* < 0.01) and 44.8% (*p* < 0.05), respectively. Next, we measured the expression of Nrf2-dependent antioxidant defense genes including HO-1, GCLC, and NQO-1. mRNA (Figure 1C) and protein expression (Figure 1D) of HO-1 and NQO-1 increased significantly after 1-MNA treatment, which similar to that observed for Nrf2. The up-regulated genes in1-MNA treatment group were1.25–12-fold increase, whereas the protein levels were increased by 0.5–1.3-fold. However, the protein expression of GCLC was almost unchanged after 1-MNA treatment. These results indicated that 1-MNA may act as an antioxidant by upregulating Nrf2 and antioxidant-related genes.

### 1-MNA Inhibited Apoptosis of PA-Induced H9C2 Cells

**Figure 2:**
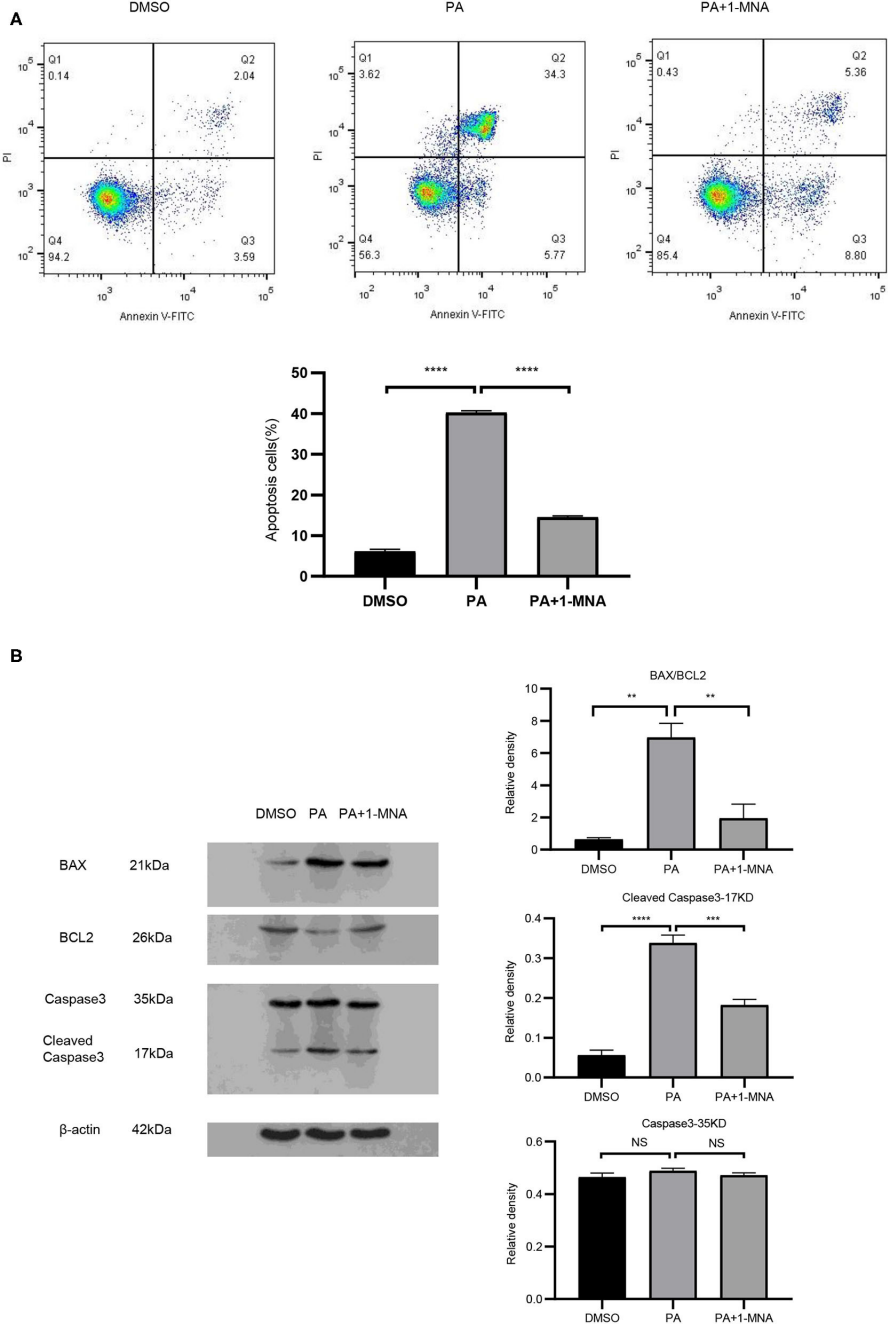
1-MNA inhibits apoptosis of PA-induced H9C2 cells. **(A)** Cell and nuclear images by flow cytometry analysis. Data are shown as mean ± SEM from three independent experiments. **(B)** Western blotting analysis of Caspase-3, Bax, and Bcl-2. The image of the gel shown is a representative picture. (*n* = 3, NS = no significance, ^**^p < 0.01, ^***^p < 0.001, ^****^p < 0.0001).

We next explored the protective effects of 1-MNA on H9C2 cells damaged by PA. Cell apoptosis were detected by Annexin V-FITC staining (Figure 2A). Flow cytometry analysis demonstrated that the apoptosis of PA treated H9C2 cells was significantly ameliorated under 10 mM 1-MNA treatment. The cells treated with PA showed an increase of Annexin V-FITC staining positive cells by 41% (vs. DMSO, *p* < 0.01). 1-MNA treatment reduced PA-induced, the apoptosis rate by 25.7% (*p* < 0.001). In the figure, the cell dots in the quadrant (upper left quadrant) where Annexin V-FITC staining is negative and PI staining is positive (Annexin V-/PI+) is the detection error within the allowable range. In this experiment apoptotic cells and necrotic cells were identified as apoptotic cells. We also examined the key pro-apoptotic protein expression. In response to the PA treatment, the proteins expression of cleaved caspase-3 and BAX/BCL2 were increased by 503.6% (*p* < 0.0001) and 989.1% (*p* < 0.01), respectively. This effects were reduced by 10 mM 1-MNA by 46.2% (*p* < 0.001) and 72.2% (*p* < 0.01; Figure 2B).

### 1-MNA Exerted Anti-inflammatory Effects on H9C2 Cells Induced by PA

**Figure 3:**
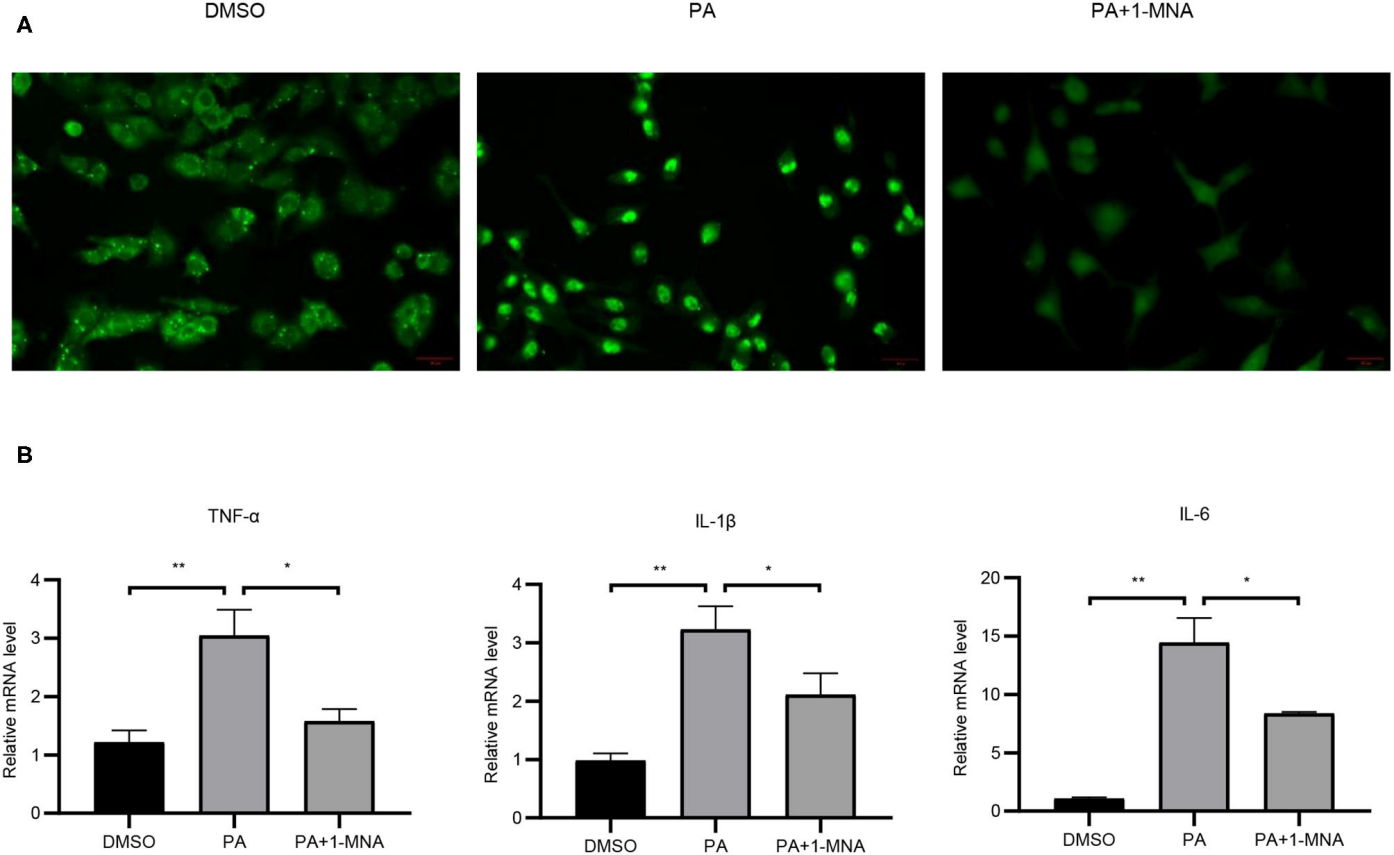
Effects of 1-MNA on PA-induced inflammation in H9C2 cells. **(A)** Immunofluorescence assay for NF-κB p65 nuclear translocation for NF-κB activity. **(B)** The mRNA expression of inflammatory cytokines. β-actin was taken as an internal control gene. Expression is expressed as the mean ± SEM. Differences in expression were analyzed by the unpaired Student′s *t*-test and one-way ANOVA (*n* = 3, NS = no significance, ^*^p < 0.05, ^**^p < 0.01).

Next we evaluated the anti-inflammatory activity of 1-MNA in H9C2 cells. To detect the expression and distribution of NF-κBp65 in H9C2 cells, we performed an immunofluorescence assay. In unstimulated cells, the majority of NF-κB resides in the cytoplasm in association with the IκB family of inhibitory molecules, which mask the nuclear localization signals of NF-κB. By PA treatment, degradation of IκB releases NF-κB to translocate to the nucleus where it binds κB enhancer elements and modulates gene expression and 1-MNA can significantly hinder this process. Figure 3A showed NFκB-p65 accumulated in the nucleus in PA-treated cells. We measured the mRNA expression of several inflammatory cytokines which were activated by NF-κB such as TNF-α, IL-1, and IL-6 (Figure 3B). PA increased the mRNA levels of TNF-α by 151.2% (*p* < 0.01), IL-1 by 228.6% (*p* < 0.01), and IL-6 1238.1% (*p* < 0.01). However, the activation of NF-κB pathway by PA was significantly inhibited in 1-MNA treated cells. 1-MNA decreased the mRNA expression of TNF-α, IL-1, and IL-6 by 48.4% (*p* < 0.05), 34.5% (*p* < 0.05) and 42% (*p* < 0.05) induced by PA.

### 1-MNA Attenuated PA-Induced Hypertrophy in H9C2 Cells

**Figure 4:**
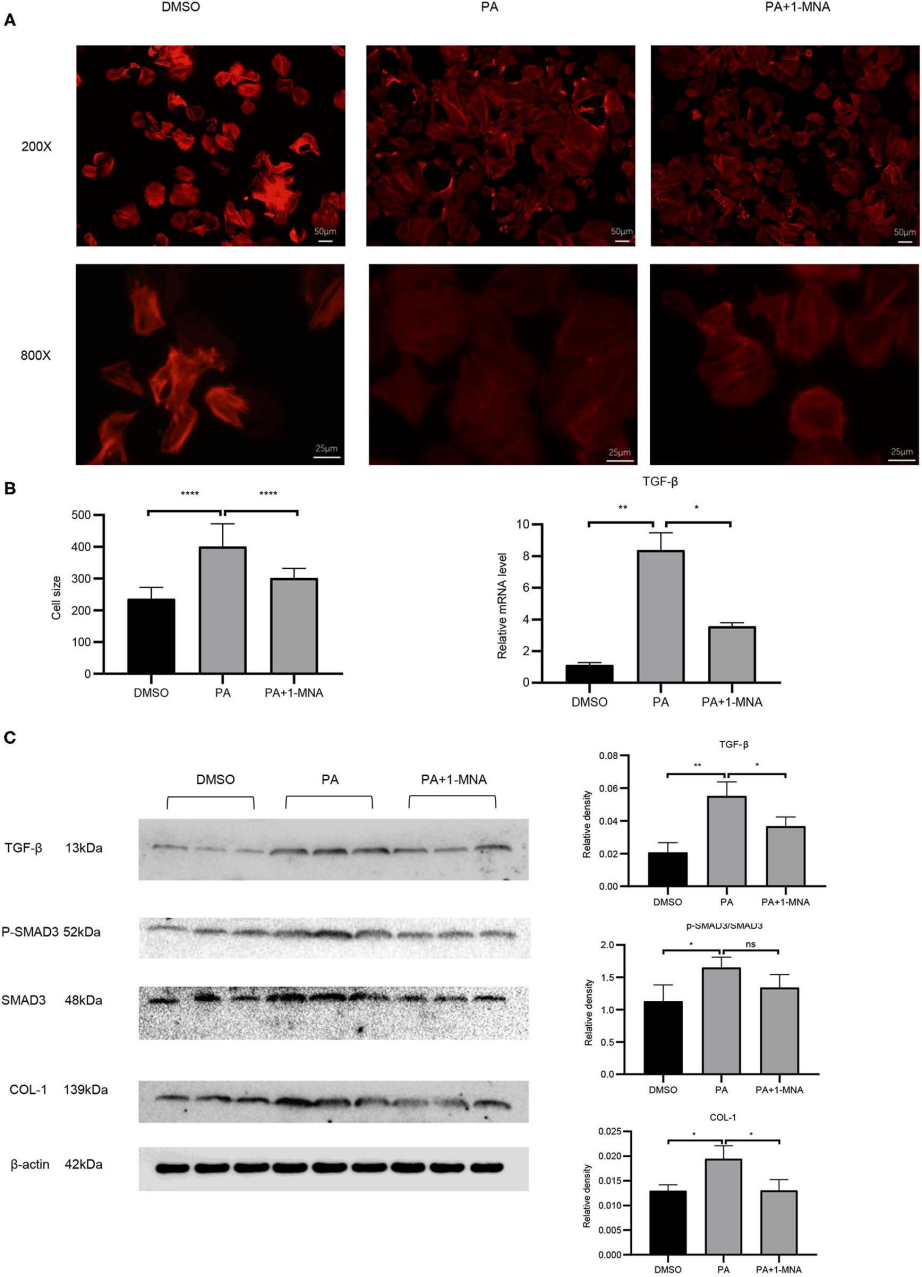
Effects of 1-MNA on PA-induced hypertrophy and fibrosis in H9C2 cells. Representative images of H9C2 cells stained with rhodamine-phalloidin from three independent experiments. **(A)** Representative staining images are shown with the quantitative data for the cell size of 100 randomly selected cells in 3 independent experiments. **(B**) The mRNA expression of TGF-β. **(C)** Western blotting analysis of TGF-β, smad2/3, p-smad2/3, and Col-1. β-actin was taken as an internal control gene. The image of the gel shown is a representative image from three independent experiments and intensity were normalized (*n* = 3, NS = no significance, ^*^p < 0.05, ^**^p < 0.01, ^****^p < 0.0001).

Cardiac hypertrophy and fibrosis are the major pathological features of chronic heart disease. We used rhodamine-phalloidin to detect the surface area of H9C2 cell (*n* = 100). As shown in Figure 4A, 1-MNA significantly inhibited hypertrophy in PA treated H9C2 cells by 25.1% (*p* < 0.0001). qRT-PCR analysis showed that the mRNA level of TGF-β (Figure 4B), a marker gene of myocardial fibrosis, was increased by 648.2% (*p* < 0.01) in the cells treated with PA. 1-MNA treatment resulted in a decrease of TGF-β by 57.5% (*p* < 0.05) induced by PA. We then examined multiple TGF-β signaling pathway related proteins. 1-MNA treatment inhibited PA-induced Col-1 (31.6% *p* < 0.05)and TGF-β (34.5% *p* < 0.05) (Figure 4C).

### 1-MNA Reduced Levels of Some Obesity Indicators in HFD-Fed Mice

**Figure 5:**
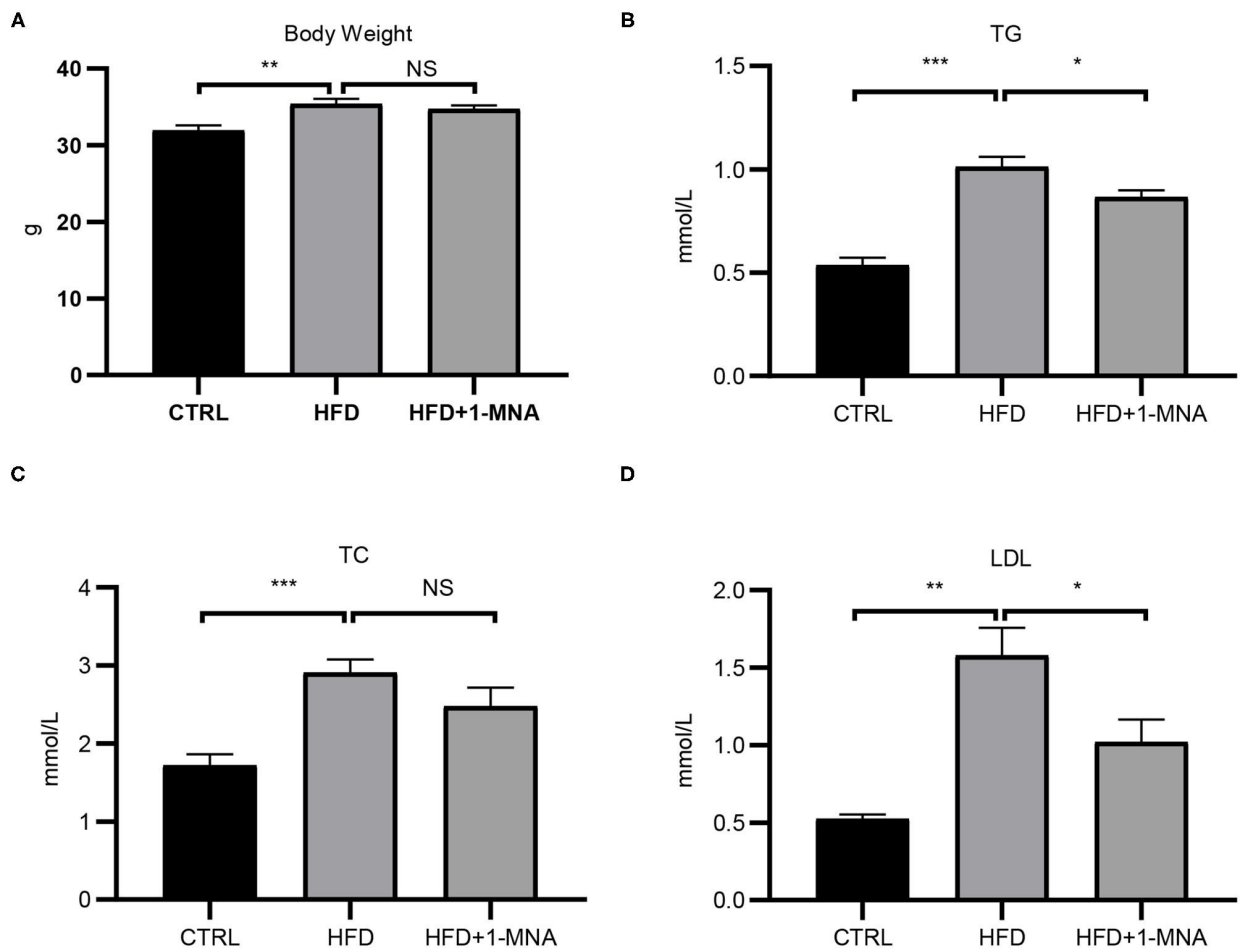
Effects on serum TC, TG, LDL, and body weight following 1-MNA treatment. HFD induces increased **(B)** serum TG and **(D)** LDL, which are inhibited by 1-MNA treatment. **(A)** Body weight and **(C)** TC were different but not statistically significant. (*n* = 3, NS = no significance, ^*^p < 0.05, ^**^p < 0.01, ^***^p < 0.001).

The HFD-induced lipotoxicity mouse model used in this study was previously reported by Tanaka et al. (10). Mice fed with HFD or fed with HFD and 100 mg/kg/day 1-MNA for 18 weeks. At the end of the experiment, the mouse body weight, TG, TC, and LDL were measured. Figure 5A showed the body weight of mice fed with HFD increased 11% (*p* < 0.01). 1-MNA administration did not significantly reduce HDF caused body weight. This could be due to relatively short period of 18 weeks experiment. Mice fed HFD showed significantly increased TG (90.6% *p* < 0.001), TC (69.2% *p* < 0.001), and LDL (195.1% *p* < 0.01) (Figures 5B–D), while mice given 1-MNA demonstrated decreased TG (14.4% *p* < 0.05) and LDL (35.5% *p* < 0.05) compared with HDF mice. TC also decreased but the results did not reach statistical significance (Figure 5C).

### 1-MNA Influenced Oxidative Stress and Inflammation in the HFD-Fed Mouse Heart

**Figure 6:**
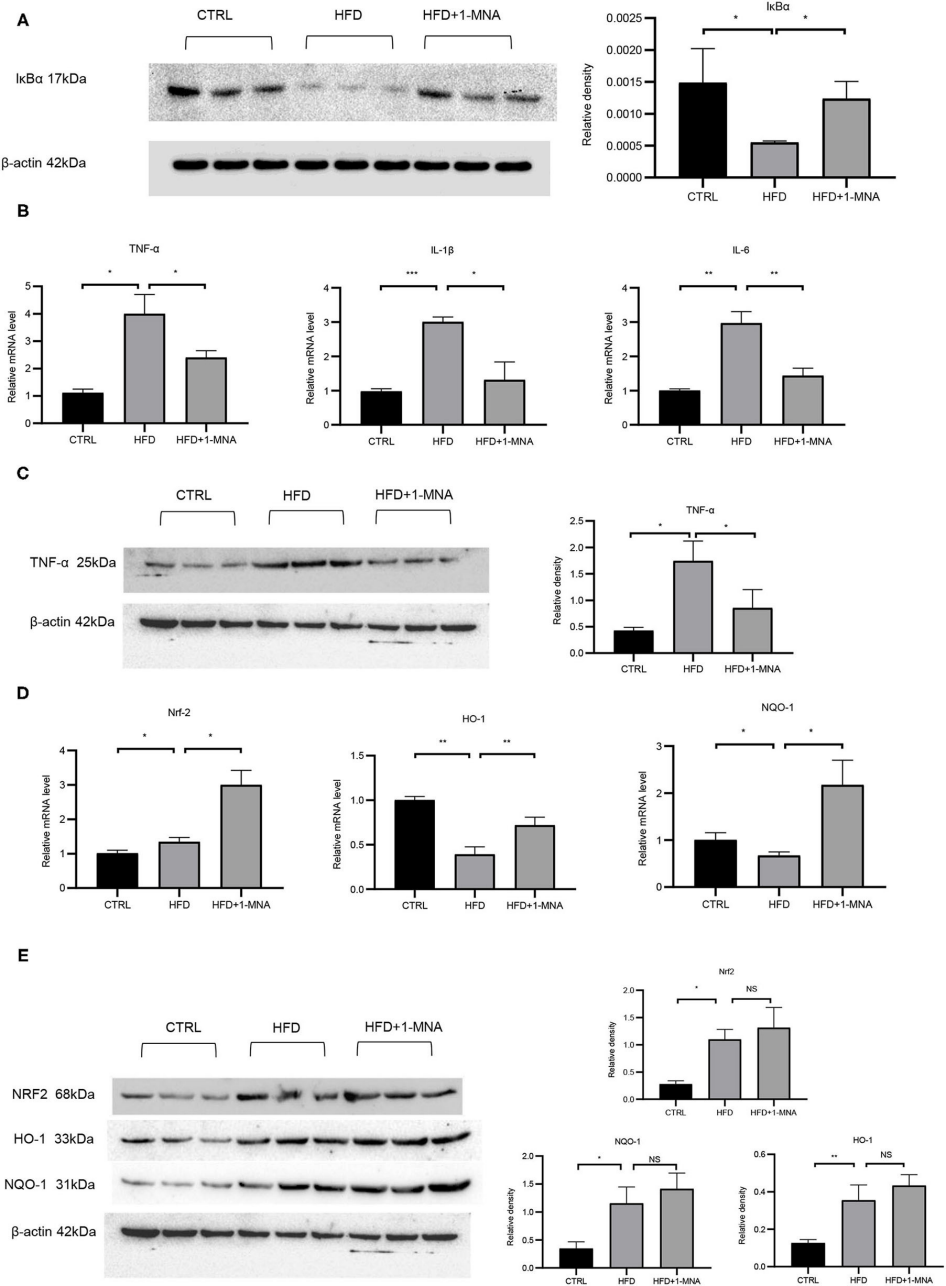
1-MNA administration improved the oxidative stress and inflammation of the heart induced by HFD. **(A)** Western blotting analysis of IκB expression. Quantification of protein expression shown is normalized to β-actin. **(B)** qRT-PCR analysis of oxidative stress related gene expression. **(C)** Western blotting analysis of TNF-α. β-actin was taken as an internal control gene. **(D)** qRT-PCR analysis of inflammation related gene expression. **(E)** Western blotting analysis of NRF2, HO-1, and NQO-1. β-actin was taken as an internal control gene. (*n* = 3, NS = no significance, ^*^p < 0.05, ^**^p < 0.01, ^***^p < 0.001).

The IκB degradation is a key step in NF-κB activation, as it allowed NF-κBp65 to be transported from the cytoplasm to the nucleus. Next, we investigated the expression of IκB at the protein level to determine whether 1-MNA inhibited the activation of NF-κB. As shown in Figure 6A, protein levels of IκB-α in heart samples of mice from the HFD-fed group were reduced by 62.8% (*p* < 0.05), while IκB-α protein levels were partially restored in mice treated with 1-MNA which is a 123.6% (*p* < 0.05) increase. mRNA expression of TNF-α, IL-1β, and IL-6 (Figure 6B), which are closely related to the activation of NF-κB, increased 2-2.6 fold (*p* < 0.05) in HFD-fed mice as compared to the untreated control. 1-MNA inhibited the expression of inflammatory cytokines induced by HFD by 40–56.2% (*p* < 0.05). TNF-α protein levels were increased by 309.6% (*p* < 0.05) in the HFD group (Figure 6C), 1-MNA administration inhibited HFD-induced TNF-α expression by 50.9% (*p* < 0.05). Next, we measured the effects of 1-MNA on oxidative stress in mouse hearts. Figure 6D showed the increased expression of Nrf2 in HFD-fed mice heart tissue samples, reflecting a relative increase of 32.6%. 1-MNA treatment increased Nrf2 mRNA expression by 55.2% (*p* < 0.05) in comparison to the HFD group. However, the elevated protein expression was not statistically significant. There was an elevation of 294.1% (*p* < 0.05) in the HFD group and no significant change in expression after 1-MNA treatment (Figure 6E). Consistently, the mRNA expression of the downstream antioxidant genes, such as HO-1 and NQO-1 were decreased in HFD group, which were decreased at 60.9% (*p* < 0.05) and 32.7% (*p* < 0.01), respectively. After 1-MNA treatment, the mRNA expression of HO-1 and NQO-1 were increased 84.1% (*p* < 0.05) and 223.3% (*p* < 0.01) (Figure 6D). In response to the HFD treatment, the proteins expression of HO-1 and NQO-1 were increased by 180.8% (*p* < 0.01) and 232.6% (*p* < 0.05), respectively, but similarly, no significant changes were reflected in the HFD+1-MNA group (Figure 6E).

### 1-MNA Induced Anti-fibrotic Effects in the Mouse Heart

**Figure 7:**
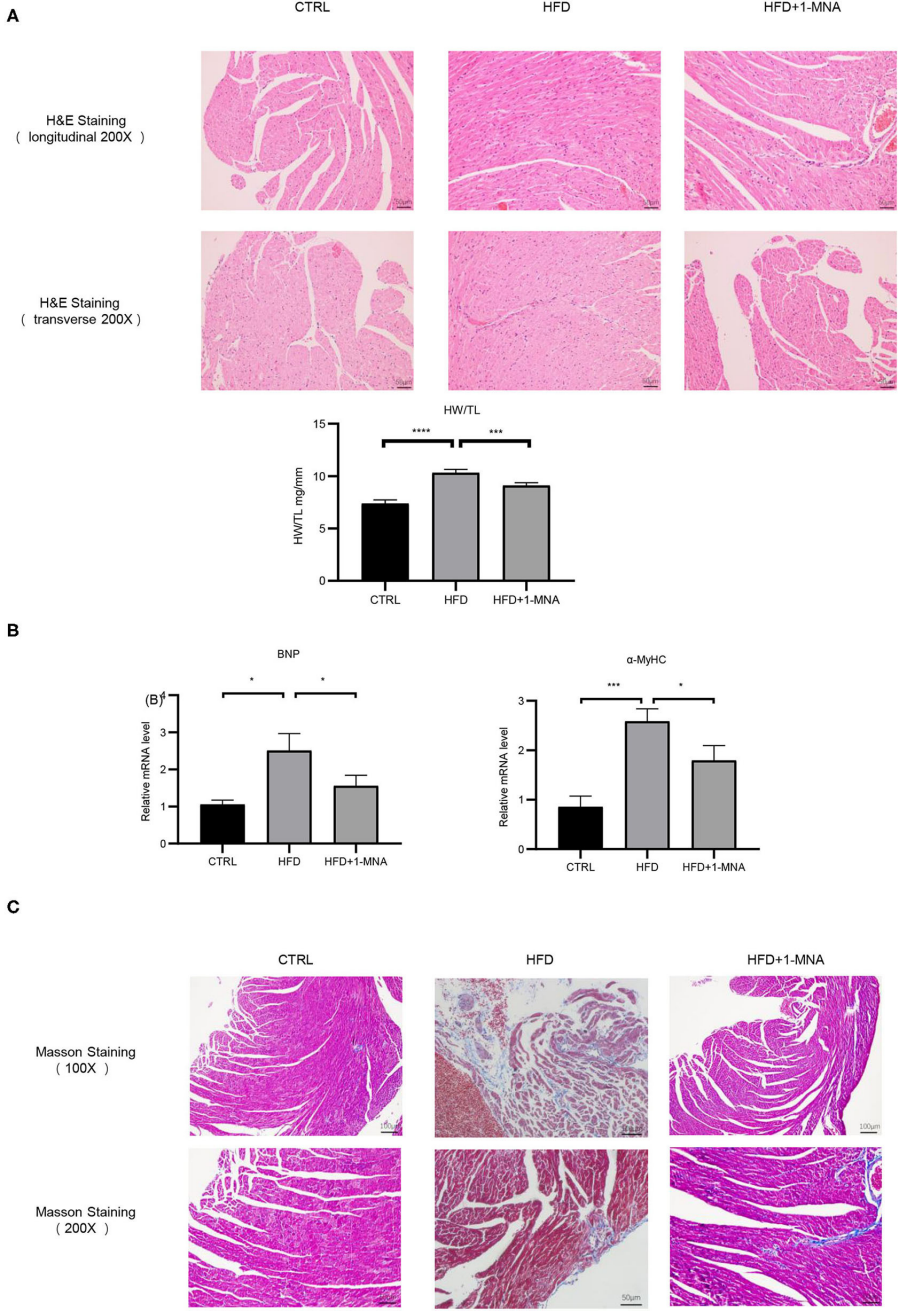
1-MNA amileorated HFD-induced heart injury. **(A)** Representative images from longitudinal and transverse H&E staining of heart tissues are shown. Data for the ratio of heart weight (HW) to tibia length (TL). **(B)** 1-MNA administration reduced the indicated gene expression in HFD-induced hearts. **(C)** Myocardial tissue was colored in red and collagen was colored in blue by Masson staining. Under light microscopy, the myocardial fibers of the control group were arranged neatly, with no obvious breaks or rearrangements. The size of the intercellular space was normal. (*n* = 5, ^*^p < 0.05, ^***^p < 0.001, ^****^p < 0.0001).

Next, we assessed the histological changes of mouse cardiac tissue. HFD-fed mice showed myocardial fiber fracture and cell morphological abnormalities. 1-MNA administration alleviated cardiac damage caused by HFD (Figure 7A). The tibia length will no longer change after the mouse matures and can be used as a quantitative comparison standard. We measured the heart weight/tibia length (HW/TL) ratio. Compared with controls, growth of HW/TL increased by 39.7% (*p* < 0.0001) on week 18 in HFD mice. After 1-MNA treatment, the HW/TL ratio was reversed by 11.7% (*p* < 0.001) (Figure 7A). Two cardiac hypertrophy marker genes, BNP and α-myosin heavy chain, were analyzed by real-time PCR. 1-MNA treatment reduced HFD-induced BNP(37.7% *p* < 0.05) and α-myosin heavy chain(30.8% *p* < 0.05) gene expression (Figure 7B). These results further indicate that 1-MNA has anti-fibrosis and hypertrophy function in the treatment of heart injury induced by hyperlipidemia. The structure change of the myocardial tissue was further examined by Masson's trichrome staining (Figure 7C). The results showed that the myocardial fibers in the HFD group were disordered and dissolved, the intercellular space was widened, and the deposition of collagen fibers increased. Thses morphological alteration was improved after 1-MNA treatment. The collagen volume fraction in the HFD group was significantly higher than the control group, while the 1-MNA treated mice have a lower collagen volume fraction than the HFD group.

**Figure 8:**
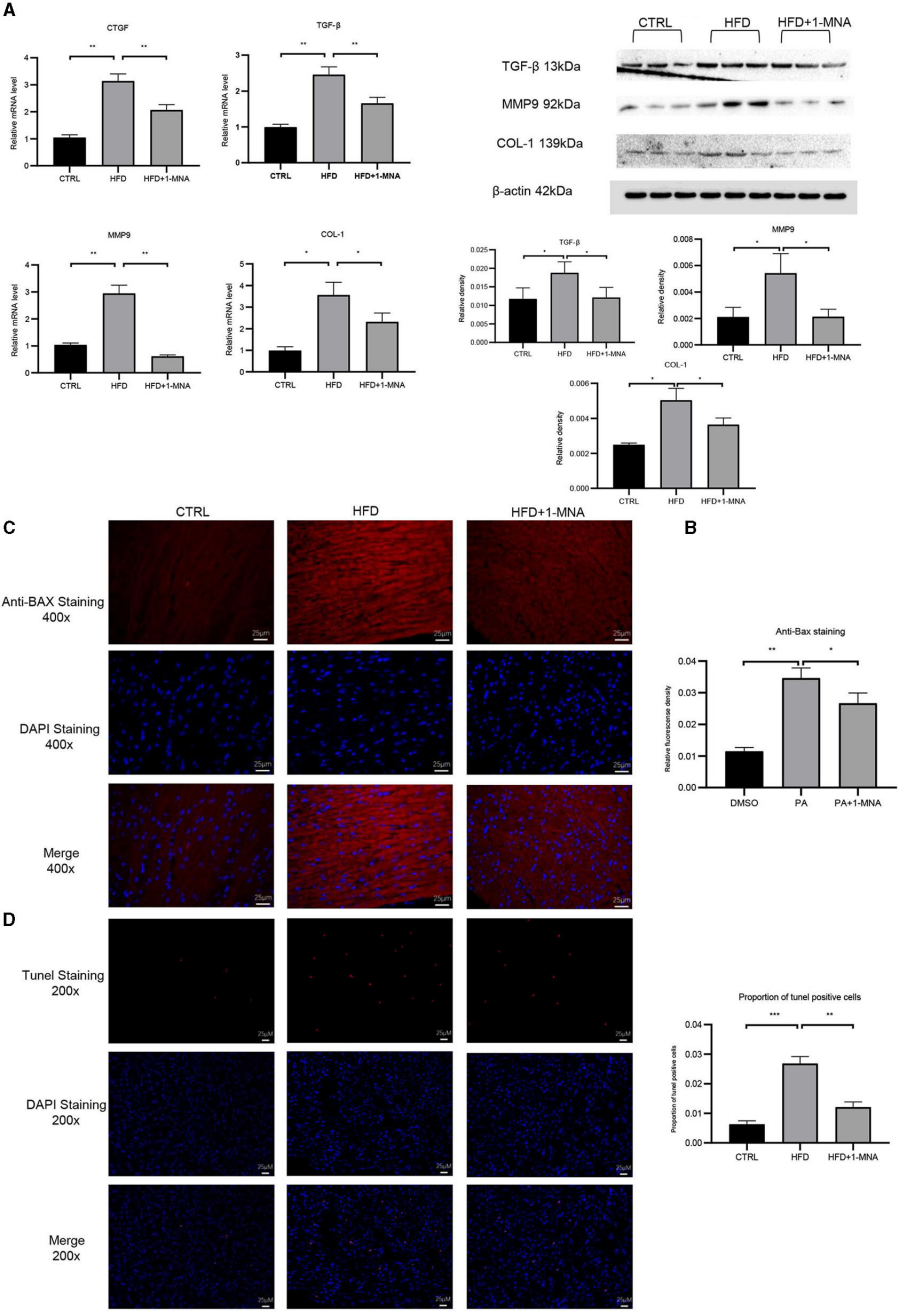
1-MNA administration improved the fibrosis and apoptosis of the heart induced by HFD. **(A)** Heart tissues from each group were individually processed for RNA extraction and qRT-PCR analysis. **(B)** Western blotting analysis of TGF-β, MMP9, and COL-1.1-MNA administration inhibited HFD-induced cardiomyocyte apoptosis. Images of cardiac tissue sections subject to immunohistochemical staining for Bax. **(C)** The histogram shows the relative fluorescence density of anti-Bax staining. **(D)** Images of cardiac tissue sections subject to immunohistochemical staining for Tunel. (*n* = 5, ^*^p < 0.05, ^**^p < 0.01, ^***^p < 0.001).

qRT-PCR analysis showed an increase of 200.8% (*p* < 0.01) of CTGF and 147.8% (*p* < 0.01) of TGF-β expression in the HFD group (Figure 8A). After 1-MNA treatment, the expression of CTGF and TGF-β were downregulated 34.2 and 32.7% (*p* < 0.01) (Figures 6A,B), and the deposition of collagen was also ameliorated. We further detected the mRNA expression level of extracellular proteins including collagen 1 and MMP-9 in total RNA from heart tissue (Figures 8A,B). These two genes expression were elevated 260.2% (*p* < 0.05) and 185.1% (*p* < 0.01). The genes expression was decreased 35% (*p* < 0.05) and 79.3% (*p* < 0.01) in the HFD+1-MNA group compared to the HFD group. Similar results were observed at the protein level (Figure 8B), TGF-β, MMP9 and COL-1 were increased by 63.6–157.1% (*p* < 0.05) in HFD group. 1-MNA administration reduced TGF-β,MMP9 and COL-1 levels by 27.8–60.9% (*p* < 0.05) as compared to the HFD group.

### Anti-apoptotic Effects of 1-MNA in Cardiomyocytes

Finally, we investigated the anti-apoptotic effects of 1-MNA. We observed a significant increase (209.1%, *p* < 0.01) in the pro-apoptotic gene BAX in heart tissue of mice fed a HFD. 1-MNA effectively prevented HFD-induced BAX by 23.1% (Figure 8C). Tunel staining experiment (Figure 8D) showed that the number of positive cells was increased by 332.3% (*p* < 0.001) in HFD group. 1-MNA treatment significantly reduced TUNEL positive cells by 55.2%. Parallel to our *in vitro* study, administration of 1-MNA to HFD-fed mice effectively induced activation of Bax and reduced the ratio of Tunel positive cells; thus, the anti-apoptotic effects of 1-MNA may be associated with the inhibition of inflammation and oxidative stress.

## Discussion

Hyperlipidemia is caused by high serum levels of TC, TG, and LDL cholesterol, or low levels of circulating high-density lipoprotein cholesterol (12). Hyperlipidemia is a disorder of systemic lipid metabolism, and is one of the main risk factors for inducing atherosclerosis, fatty liver, and cardiovascular and cerebrovascular diseases (13, 14). Hyperlipidemia consists of hypertriglyceridemia, hypercholesterolemia, and mixed hyperlipidemia, in which both TG and TC are elevated. High levels of TGS and TC, are accompanied by a large amount of FFA and LDLs in the blood, are the most essential features of hyperlipidemia. Our research also confirmed this (Figures 5B,D). Diseases such as stroke, atherosclerosis, and coronary heart disease are closely related to hyperlipidemia (15). FFA and its metabolite TG cause cell damage, which is called lipotoxicity. Studies have shown that the leading cause of liver lipotoxicity is FFA rather than TGs. In hyperlipidemia, the increase in plasma FFA is the most important feature. It has become the main cause of obesity-related diseases such as coronary heart disease and atherosclerosis (16). According to our research,we clarified the preventive effects of 1-MNA on cardiac histopathological changes, the oxidative stress response, inflammation, and cell death pathways in the HFD mouse model and PA-treated cardiomyocytes.

Adipokines are mainly secreted by adipose tissue and can regulate glucose and lipid metabolism, inflammation, immune response, and cardiovascular function (17, 18). The relationship between obesity and cardiovascular disease has been confirmed in clinical and experimental models (19, 20). Thus, there is an urgent need to clarify the mechanism of hyperlipidemia-induced heart damage and to discover new therapeutic agents. 1-MNA, a primary metabolite of NNMT enzyme catalyzed the methylation of nicotinamide, plays an important roles in inflammatory response and immune function. We first demonstrated that 1-MNA is spontaneously secreted and acts in PA-induced H9C2 cells through work such as literature review and pre-experiments (Figure 1A), whereby we next explored the possible antioxidant role of 1-MNA in a model of PA-induced cardiomyocyte injury.

PA is the main saturated FFA in plasma, which stimulates the production of ROS in endothelial cells and smooth muscle cells to promote the expression of inflammatory cytokines (21). In our experiments, PA treatment in H9C2 cells clearly showed an increase in ROS stress. Many studies have confirmed that Nrf2 is involved in the control of many oxidative stress-related genes (22, 23). Nrf2 activates antioxidant factors such as NQO1, HO-1, SOD, and CAT, etc., and also regulates the GSH redox system to inhibit oxidation. Our results showed that 1-MNA treatment increased the expression of Nrf2 *in vitro* (Figures 1C,D). Further, 1-MNA treatment also significantly increased the expression of Nrf2 downstream genes GCLC, HO-1, and NQO-1 (Figures 1C,D).

Although several studies (24, 25) have revealed the mechanisms involved in 1-MNA's antioxidant effects, our observations differed slightly from those of a previous report by Tanaka et al. (10), which found that 1-MNA treatment did not influence the increase in PA-induced antioxidant enzymes such as manganese superoxide dismutase and HO-1 mRNA levels. 1-MNA treatment improved PA-induced apoptosis and necrosis by inhibiting mitochondrial oxidative stress in PTCs (Proximal tubule cells), without influencing the mRNA levels of antioxidant enzymes or the intracellular concentrations of NAD or NADH. Some potential reasons for these differences include: (1) different cell types used in the studies, (2) unique cell culture conditions, and signaling molecules, (3) and the source of 1-MNA.

Recently, it has been reported that 1-MNA improves oxidative stress and cell death of proximal renal tubular cells which is caused by lipid toxicity (10). Furthermore, 1-MNA has been shown to interact with inflammatory mediators and regulate the inflammatory response in tissues (26), but the role of 1-MNA in patient heart with hyperlipidemia remains to be explored. We found significantly improved pathological indicators of HFD-induced heart damage after 1-MNA treatment. The administration of 1-MNA at 100 mg/kg/day ameliorated the characteristics of HFD-induced hyperlipidemia (Figure 5), indicating that one of the effects of 1-MNA on the HFD-fed mice heart is to decrease the blood lipid levels.

Chronic inflammation is closely related to obesity and it has been confirmed that obesity-related chronic inflammation is a high-risk factor for cardiovascular disease (27). Hyperlipidemia promotes the production of a variety of inflammatory molecules, which aggravate the inflammatory response. In macrophages, FFA triggers inflammation by activating Toll-like receptor 4, peroxisome proliferator receptor, and inflammatory gene expression by NF-κB (28). NF-κB is a key transcription factor that regulates inflammation, and is mainly composed of two subunits, p50 and p65. NF-κB is activated in response to cytokines, pathogens, and radiation. Our study showed that PA increases p65 translocation and NF-κB activity in cultured cardiomyocytes, while HFD reduces IκB-α levels in heart tissue of mice. Further, 1-MNA significantly inhibited PA/HFD-induced NF-κB activation, thereby reducing the expression of inflammatory cytokines such as TNF-α, IL-6, and IL-1 *in vitro* and *in vivo*. The results indicated that 1-MNA may suppress PA-induced cardiac inflammation by inhibiting NF-κB.

The occurrence and development of cardiac hypertrophy are closely related to oxidative stress. The expression of α-MyHC and BNP are considered molecular markers of cardiac hypertrophy (29), which we confirmed were upregulated in our study. Moreover, 1-MNA treatment attenuated α-MyHC- and BNP-induced cardiac hypertrophy in HFD-fed mice. Pathological cardiac hypertrophy is caused by the accumulation of collagen and the expansion of the ECM due to inflammation. As a significant feature of obesity, oxidative stress participates in the formation of fibrosis by activating TGF-β (30). We observed a wide range of fibrotic reactions in HFD-fed mice hearts, which was confirmed by the increase in mRNA expression of TGF-β, CTGF, collagen 1, and MMP-9. The antagonism of 1-MNA to fibrosis was confirmed *in vivo* and *in vitro*.

Our experiment also had many unsatisfactory problems, such as the low number of animals in some of the in *vivo* experiments. Because of some objective unavoidable factors, in the Western Blot experiment *in vivo*, only 3 mice were left for experiments. In terms of statistics, *n* ≥ 3 does have a small sample size. However, combined with the cell experiments we think our experimental results still have some credibility, the small sample *t*-test is still barely usable. This is a inadequacy of this paper, and we will continue the relevant research in the future that will expand the sample size of each group to make the conclusions more convincing and will systematically explore the potential role of 1-MNA in heart disease.

**Figure 9:**
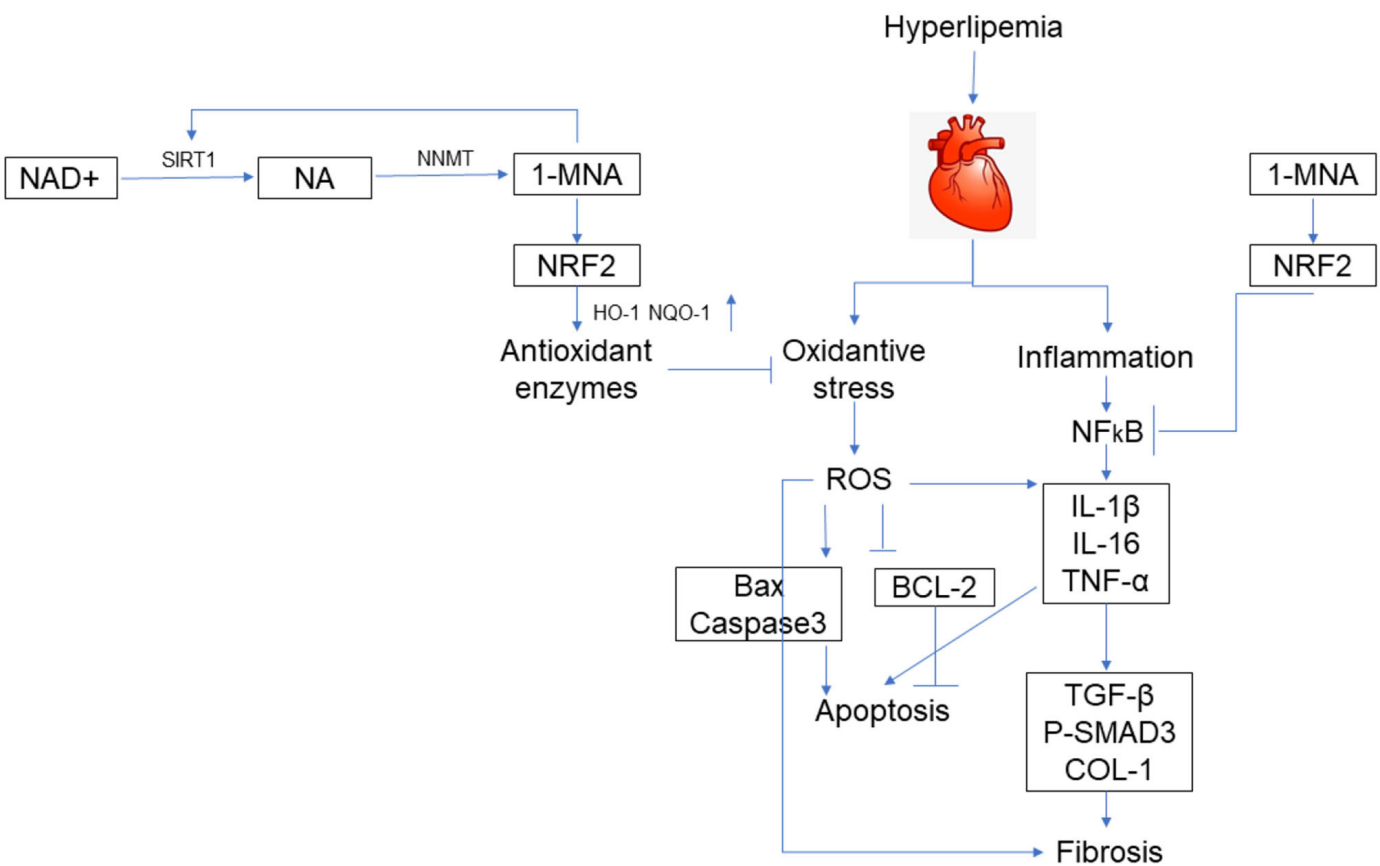
Schematic illustration. A schematic illustration for the prevention of 1-MNA from FFA/HFD-induced injury in cardiomyocytes and hearts.

In conclusion, our study confirmed that the myocardium of the HFD mouse animal model presents evidence of chronic fibrosis. TGF-β and CTGF expression in the tissue of HFD-fed mice hearts increased, which subsequently induced the expression of ECM proteins such as collagen 1. Our study showed that following 1-MNA treatment, these fibrosis indices were all downregulated, which revealed the anti-fibrotic ability of 1-MNA in HFD-fed mice hearts. High fat-induced inflammation, apoptosis, fibrosis, and hypertrophy were improved by 1-MNA both *in vivo* and *in vitro*. These effects of 1-MNA are closely dependent on its ability to increase Nrf2 expression and inhibit NF-κB activity (Figure 9). 1-MNA may inhibit TGF-β collateralization by activating SIRT1. Moreover, our study explored the regulatory effects of Nrf2 and NF-κB on hyperlipidemia-induced cardiac injury, through which we shed light on its protective role in cardiac hypertrophy-induced oxidative stress and PA-stimulated inflammation. Unfortunately, we did not use pathway inhibitors in the current study, and in future studies we will add inhibitors to explore the specific mechanisms by which 1-MNA acts. In conclusion these results suggest that Nrf2, NF-κB and SIRT1 may be important targets for the treatment of myocardial injury and related diseases caused by hyperlipidemia.

## Data Availability Statement

The raw data supporting the conclusions of this article will be made available by the authors, without undue reservation.

## Ethics Statement

The animal study was reviewed and approved by the Fourth Affiliated Hospital of Harbin Medical University Ethics Committee.

## Author Contributions

XS and ZiS designed the experiments. ZiS, PG, XZ, and ZN carried out the experiments. ZiS, ML, and ZhS provided reagents, materials, and analysis tools. ZiS wrote the manuscript. XS supervised the study and revised the manuscript. All authors reviewed the manuscript, contributed to its contents, and approved the submitted version.

## Funding

This research was supported by the Foundation for Innovative Research Groups of the National Natural Science Foundation of China (Grant/Award Numbers: 81300248 and 81570358), 2021 Shanghai College of Health Sciences Natural Science Key Project (Grant/Award Number: SSF-21-17-01), and Discipline construction project of Pudong New District Health and Family Planning Commission of Shanghai, China (Grant/Award Numbers: PWZxq2017-01 and PWYgy2018-03).

## Conflict of Interest

The authors declare that the research was conducted in the absence of any commercial or financial relationships that could be construed as a potential conflict of interest.

## Publisher's Note

All claims expressed in this article are solely those of the authors and do not necessarily represent those of their affiliated organizations, or those of the publisher, the editors and the reviewers. Any product that may be evaluated in this article, or claim that may be made by its manufacturer, is not guaranteed or endorsed by the publisher.

## Abbreviations

FFA: free fatty acids
NAD: nicotinamide adenine dinucleotide
NNMT: nicotinamide N-methyltransferase
PA: palmitic acid
1-MNA: 1-methylnicotinamide
HFD: high-fat diet
SIRT1: silent information regulator 1
SAM: S-adenosylmethionine
TG: Triglycerides
TC: Serum total cholesterol
LDL: Low-density lipoprotein.
